# Spexin-Based Galanin Receptor Type 2 Agonist for Comorbid Mood Disorders and Abnormal Body Weight

**DOI:** 10.3389/fnins.2019.00391

**Published:** 2019-04-18

**Authors:** Seongsik Yun, Arfaxad Reyes-Alcaraz, Yoo-Na Lee, Hyo Jeong Yong, Jeewon Choi, Byung-Joo Ham, Jong-Woo Sohn, Dong-Hoon Kim, Gi Hoon Son, Hyun Kim, Soon-Gu Kwon, Dong Sik Kim, Bong Chul Kim, Jong-Ik Hwang, Jae Young Seong

**Affiliations:** ^1^Graduate School of Medicine, Korea University, Seoul, South Korea; ^2^Graduate School of Medical Science and Engineering, Korea Advanced Institute of Science and Technology, Daejeon, South Korea; ^3^Department of Psychiatry, College of Medicine, Korea University, Seoul, South Korea; ^4^Neuracle Science Co., Ltd., Seoul, South Korea

**Keywords:** galanin receptor 2 agonist, depression, post-traumatic stress disorder, body weight, appetite, intranasal administration

## Abstract

Despite the established comorbidity between mood disorders and abnormal eating behaviors, the underlying molecular mechanism and therapeutics remain to be resolved. Here, we show that a spexin-based galanin receptor type 2 agonist (SG2A) simultaneously normalized mood behaviors and body weight in corticosterone pellet-implanted (CORTI) mice, which are underweight and exhibit signs of anhedonia, increased anxiety, and depression. Administration of SG2A into the lateral ventricle produced antidepressive and anxiolytic effects in CORTI mice. Additionally, SG2A led to a recovery of body weight in CORTI mice while it induced significant weight loss in normal mice. In Pavlovian fear-conditioned mice, SG2A decreased contextual and auditory fear memory consolidation but accelerated the extinction of acquired fear memory without altering innate fear and recognition memory. The main action sites of SG2A in the brain may include serotonergic neurons in the dorsal raphe nucleus for mood control, and proopiomelanocortin/corticotropin-releasing hormone neurons in the hypothalamus for appetite and body weight control. Furthermore, intranasal administration of SG2A exerted the same anxiolytic and antidepressant-like effects and decreased food intake and body weight in a dose-dependent manner. Altogether, these results indicate that SG2A holds promise as a clinical treatment for patients with comorbid mood disorders and abnormal appetite/body weight.

## Introduction

Mood disorders such as anxiety and depression are often associated with abnormal eating behaviors, leading to either obesity or a poor diet (Simon et al., 2006), and endocrine and metabolic conditions are both exacerbated in major depression (Simon et al., 2006; Marijnissen et al., 2011). Stress can promote either an increased consumption of palatable and rewarding foods leading to obesity or a diminished appetite causing weight loss (Adam and Epel, 2007), and individuals who are underweight or obese are at a high risk for depression and anxiety (Carey et al., 2014). Therefore, mood disorders and abnormal appetite are reciprocally linked (Kloiber et al., 2007). This link has also been observed in animal models of mood disorders (Sharma et al., 2013), suggesting that a common signaling pathway underlies these phenotypes in humans and animals.

Recently, the novel neuropeptide spexin (SPX) and its receptors, galanin (GAL) receptor type 2 (GAL_2_ receptor), and type 3 (GAL_3_ receptor) (Kim et al., 2014; Yun et al., 2014), have gained attention for their possible involvement in the bidirectional regulation of mood and feeding behaviors. The function of SPX in feeding behavior is interesting, because it opposes the orexigenic function of GAL, a paralogous neuropeptide (Yun et al., 2015; Furlong and Seong, 2017). Whereas the SPX administration leads to weight loss in diet-induced obese rodents (Walewski et al., 2014) and decreased food intake in goldfish (Wong et al., 2013), GAL leads to an increase in food intake (Karatayev et al., 2009). *SPX* mRNA levels are markedly decreased in the fat tissues of obese humans (Walewski et al., 2014), whereas the circulating GAL levels, along with neuropeptide Y and leptin, are significantly higher in obese women (Baranowska et al., 1997). These differences likely reflect the activities of the targeted receptors: SPX binds with high affinity to GAL_2_ receptor and GAL_3_ receptor but not GAL_1_ receptor, whereas GAL has high potencies for GAL_1_ receptor and GAL_2_ receptor but a low potency for GAL_3_ receptor (Kim et al., 2014). In addition, the GAL_2_ receptor-mediated downstream signaling induced by SPX and GAL differs. Whereas GAL exerts both G_q_- and β-arrestin-mediated signaling of GAL_2_ receptor, SPX shows a biased agonism favoring G-protein-mediated signaling (Reyes-Alcaraz et al., 2018).

When exogenously administered to rodents, GAL increases their immobility in the forced swim test (FST), suggesting an increase in depression-like behavior (Kuteeva et al., 2008). Studies using peptidergic or non-peptidergic compounds with selectivity for GAL receptor subtypes suggested that GAL_1_/GAL_3_ receptor-mediated signaling contributes to the prodepressive effect, whereas GAL_2_ receptor-mediated signaling exerts antidepressive effects (Webling et al., 2012). For instance, M617, an agonist of GAL_1_ receptor and GAL_2_ receptor (GAL_1_ receptor > GAL_2_ receptor), induces depression-like behavior, but the non-peptidergic GAL_3_ receptor antagonist SNAP37889 decreases anxiety- and depression-like behaviors (Swanson et al., 2005). AR-M1896 (Gal2-11), an agonist of GAL_2/3_ receptor (GAL_2_ receptor > GAL_3_ receptor), suppresses depression-like behaviors (Webling et al., 2012), but GAL_2_ receptor knockout mice and GAL_2_ receptor antagonist M871 injected mice exhibited anxiety- and depression-like behaviors (Bailey et al., 2007). Thus, steering SPX action through GAL_2_ receptor is an optimal approach to simultaneously resolve both mood disorders and abnormal appetite/body weight.

**Figure 1:**
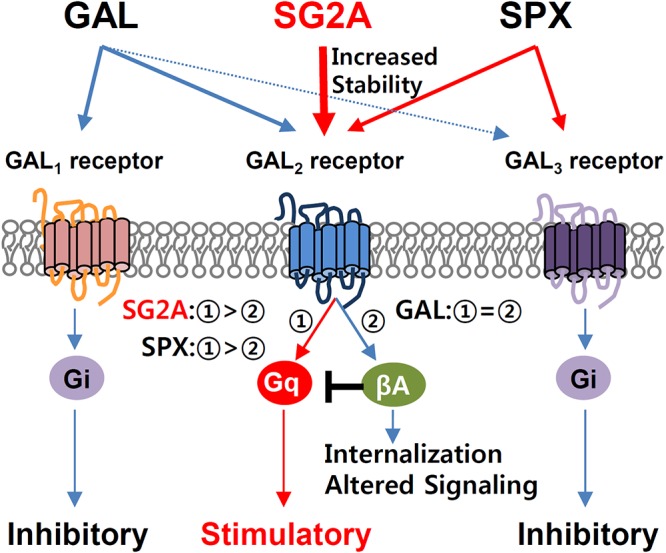
Complexity of GAL/SPX and receptor systems and pharmacological properties of SG2A. The natural ligand GAL binds to GAL_1_ receptor and GAL_2_ receptor with high affinity but shows relatively low affinity to GAL_3_ receptor. SPX has high potency to activate GAL_2_ receptor and GAL_3_ receptor but does not activate GAL_1_ receptor (Kim et al., 2014). The synthetic ligand SPX-based GAL_2_ receptor agonist (SG2A) exhibits exclusive selectivity toward GAL_2_ receptor but not GAL1 receptor and GAL_3_ receptor (Reyes-Alcaraz et al.,2016). GAL receptors have a wide range of physiological functions through subtype-specific signaling pathways. Upon stimulation by a ligand, GAL_1_ receptor and GAL_3_ receptor induce inhibitory G_i_ signaling, whereas GAL_2_ receptor triggers stimulatory G_q_ signaling. The GAL-induced GAL_2_ receptor activation induces G_q_-coupled signaling followed by the β-arrestin (βA)-mediated pathway, including receptor desensitization/internalization and initiation of an alternative signaling. Thus, GAL behaves as a classical agonist equally inducing G_q_-coupled and β-arrestin-mediated pathways. Unlike GAL, SPX and SG2A exhibit a biased GAL_2_ receptor agonism toward the G_q_-coupled pathway over β-arrestin-mediated pathway such that they induce receptor internalization much less than GAL (Reyes-Alcaraz et al., 2018).

We recently developed SPX-based GAL_2_ receptor-selective agonists (SG2A) with a much longer half-life in serum than wild-type SPX (Figure 1; Reyes-Alcaraz et al., 2016, 2018). In addition, SG2A, like SPX but unlike GAL, preferentially induced G-protein-mediated signaling over β-arrestin-dependent pathway (Reyes-Alcaraz et al., 2018), which avoids the drug tolerance or possible adverse side effects associated with classical agonists (Hausdorff et al., 1990; Yang and Tao, 2017). In the present study, we examined the beneficial effects of intracerebroventricularly (i.c.v.) administered SG2A on body weight and mood changes in mouse models of depression and anxiety disorder and investigated the putative neural networks activated by SG2A. We also examined whether intranasal (i.n.) administration of SG2A produces effects similar to those with i.c.v. administration, which would increase its potential clinical application for patients with comorbid mood disorders and abnormal body weight.

## Materials and Methods

### Animals

Wild-type male C57BL/6J mice at 8-weeks old were obtained from Central Lab Animal (Seoul, South Korea) and proopiomelanocortin (POMC)-hrGFP mice were from Jackson Laboratory (#006421; Bar Harbor, ME, United States). All mice were kept in temperature-controlled (22–23°C) rooms under a 12-h light-dark photoperiod. Standard mouse chow/water were available *ad libitum*. All animal procedures were approved by the Institutional Animal Care and Use Committee of Korea University (KOREA-2016-0050-C2) and Korea Advanced Institute of Science and Technology (KA2014-02).

### Drug Application

For i.c.v. injections, the mice were anesthetized with a flow anesthesia system, mounted on a stereotaxic apparatus (Stoelting, Wood Dale, IL, United States), and unilaterally implanted with 26-gauge stainless steel guide cannulae (model C315G; Plastics One, Roanoke, VA, United States) in the lateral ventricles [(LV) AP, -0.55 mm; ML, 1.1 mm; and DV, -2.05 mm]. A 32-gauge dummy cannula was inserted into guide cannula to prevent clogging. Two jewelry screws were implanted into the skull as anchors, and the whole assembly was affixed to the skull with dental cement. Mice were allowed to recover for 2 weeks, singly housed. SG2A (PEG2-NWTdANAALYLFGPdQ-NH_2_; AnyGen, Gwangju, South Korea) was dissolved in dimethyl sulfoxide (DMSO) to a concentration of 100 μM (170 ng/μl) and injected into the LV (0.5 μl/ventricle) 2–3 h before behavioral tests or 1–2 h before c-fos induction sampling with 33-gauge injector cannulae attached to 10-μl Hamilton syringes at a rate of 0.5 μl/min. For i.n. administration, a 10 mM stock solution of SG2A dissolved in DMSO was diluted with phosphate-buffered saline (PBS) to 1, 3, and 10 μg in 5 μl final volume, and 2.5 μl of this solution was delivered to each nostril with a pipette.

### Behavioral Studies

Mice were handled for 5 min daily for 3 days before the behavioral tests. Recorded videos were analyzed with an ANY-maze video tracking program (Stoelting).

See Supplementary Information for body weight and food intake measurements, elevated plus maze test (EPMT), open filed test (OFT), tail suspension test (TST), the FST, sucrose preference test (SPT), Pavlovian fear conditioning, unconditioned innate fear response, Y-maze test, and novel object recognition (NOR) test.

### Corticosterone Pellet Implantation (CORTI) for the Depression Model

CORTI was performed as previously described (Demuyser et al., 2016). Mice were randomly assigned to two groups (Sham or CORTI). For the CORTI group, mice were anesthetized with a flow anesthesia system and two slow-release corticosterone pellets (21-day release, 5 mg/pellet; Innovative Research of America, Sarasota, FL, United States) were subcutaneously implanted in the neck to provide an equivalent total exposure of approximately 20 mg/kg/day corticosterone depending on the weight. Sham group, were treated equally but were not implanted. Cannulae for i.c.v. were then implanted into the LV of animals in both groups. Body weights were recorded every 3 days and every week.

### Immunohistochemistry

Animals were perfused with 4% paraformaldehyde in PBS, and isolated brains were postfixed in the same fixative overnight. The brains were then cryoprotected in 30% sucrose, sectioned serially on a cryostat (40 μm), and stored in 50% glycerol/50% PBS at -20°C until use. Sections were blocked with 10% horse serum and 0.3% Triton X-100 for 30 min. Sections were incubated overnight at 4°C with primary antibodies against c-fos (Cell Signaling Technology, Danvers, MA, United States), CamKIIα (Millipore, Burlington, MA, United States), GAD67 (Millipore), tryptophan hydroxylase [(TPH) Sigma-Aldrich, St. Louis, MO, United States], tyrosine hydroxylase [(TH) Sigma-Aldrich], NeuN (Millipore), or corticotropin-releasing hormone [(CRH) Peninsula Laboratories, San Carlos, CA, United States]. After several washes with PBS, appropriate secondary antibodies, Alexa 488 and Cy3 with DAPI, were applied for 30 min. The sections were washed, mounted, and observed under a confocal microscope (Leica TCS SP8; Leica Microsystems, Buffalo Grove, IL, United States). For analysis of neurogenesis, mice were administered BrdU [100 mg/kg i.p.; 97% (+)-5′-bromo-2′-deoxyuridine; Sigma-Aldrich] twice per day (8-h interval) for 2 days prior to sacrifice. Before blocking, the brain sections were incubated with 1 N HCL for 30 min at 37°C and then incubated overnight with anti-BrdU (Abcam, Cambridge, United Kingdom) and anti-doublecortin [(DCX) Santa Cruz Biotechnology, Dallas, TX, United States] antibodies.

See Supplementary Information for Western blotting, electrophysiology and determination of α-MSH secretion in POMC neurons.

### Statistical Analysis

The data are presented as means ± standard errors of the means (SEMs) from at least two independent experiments. Statistical differences between individual groups were evaluated using Student’s *t*-tests and/or analyses of variance followed by Newman-Keuls tests as a *post hoc* comparison. Electrophysiology results were analyzed with Wilcoxon signed-rank tests. Fear memory extinction was evaluated by repeated-measures analyses of variance followed by Bonferroni tests. *P*-value of <0.05 was considered statistically significant.

## Results

### Effects of SG2A in CORTI Mice

**Figure 2:**
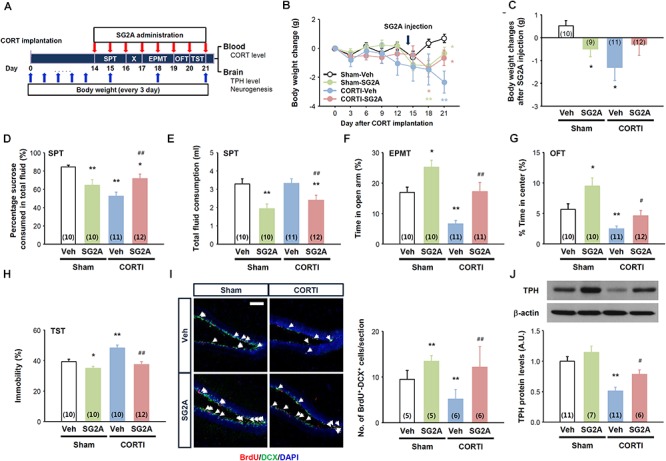
Effects of SG2A on body weight change and anxiety/depression-like behaviors in mice with corticosterone pellet implantation (CORTI) and in sham-operated mice. **(A)** Experimental scheme for SG2A administrations and behavioral tests after CORTI. SG2A or vehicle (Veh) was administered to CORTI or sham-operated mice for 8 consecutive days, during which body weight measurement, the sucrose preference test (SPT), elevated plus maze test (EPMT), open field test (OFT), and tail suspension test (TST) were conducted. After all behavioral tests, blood and brain samples were collected for determination of CORT levels, neurogenesis in the dentate gyrus of the hippocampus, and tryptophan hydroxylase (TPH) protein levels in the dorsal raphe nucleus. **(B,C)** Body weight changes during 3 weeks of CORT implantation and after SG2A administration [arrow in panel **(B)**] for 7 days. Effects of SG2A on sucrose preference **(D)**, total fluid consumption **(E)**, times in the open-arm in the EPMT **(F)** and in the center in the OFT **(G)**, and immobility of mice in the TST **(H)**. **(I)** Immunohistochemistry of BrdU (red), doublecortin [(DCX) green] and DAPI (blue) (left) and mean number of BrdU+DCX+ cells (right) in the DG of the hippocampus (5–6 slices were counted per mouse). **(J)** Western blot for tryptophan hydroxylase [(TPH) top] and TPH levels in the DRN (bottom). Data are presented as means ± SEMs; ^∗^*P* < 0.05 and ^∗∗^*P* < 0.01 vs. Sham-Veh, ^#^*P* < 0.05 and ^##^*P* < 0.01 vs. CORTI-Veh. Numbers in parentheses indicated the numbers of animals used for each group. Scale bar, 50 μm.

The effects of SG2A were first addressed in CORTI mice, which exhibit markedly lowered body weights and anhedonia-, anxiety-, and depression-like behaviors (Figure 2). The functional phenotype of these mice resembles the hypercortisolism, anhedonia, hypophagia, and weight loss in individuals with melancholic depression (Carroll et al., 2012), according to the Diagnostic and Statistical Manuals of Mental Disorders, 5th edition (American Psychiatric Association, 2013). 14 days after CORTI or sham operations, SG2A were administered i.c.v. for 8 consecutive days. During this time, the SPT was performed first for 2 days, followed by 1-day break, and then the EPMT was performed for 2 days, OFT for 1 day, and TST for 1 day. 3 to 4 h after SG2A administration on the last day, blood and brain samples were collected (Figure 2A).

High levels of blood corticosterone were maintained for 21 days in CORTI mice, regardless of SG2A administration (Supplementary Figure 1A), and the body weights of CORTI mice were significantly lower than the sham-operated/vehicle-treated (Sham-Veh) mice (Figure 2B). Notably, the effect of SG2A on body weight differed between Sham and CORTI mice. The treatment of Sham mice with SG2A (Sham-SG2A) for 7 consecutive days led to a significant body weight loss, whereas the SG2A-treated CORTI mice partly recovered the body weights (*P* = 0.08, CORTI-SG2A vs. CORTI-Veh) and did not differ from those of Sham-Veh mice (Figure 2C).

The results of the SPT suggest that CORTI induces anhedonia, a major symptom of depression, as no sucrose preference was observed in CORTI-Veh mice. SG2A administration significantly increased the sucrose preference rate in CORTI mice but induced a moderate but significant decrease in sucrose preference in Sham mice (Figure 2D). Notably, total fluid consumption did not differ between CORTI-Veh and Sham-Veh mice (Figure 2E), indicating that the decrease of sucrose preference in CORTI-Veh mice is likely due to anhedonia. By contrast, the decrease of sucrose preference in Sham-SG2A and CORTI-SG2A mice accompanied decreased total fluid (Figure 2E) and sucrose (Supplementary Figure 1) consumption.

In the EPMT, CORT-Veh mice appeared to be very anxious, as their time in the open-arms was significantly less than that of Sham-Veh mice. SG2A administration increased the time in the open-arms for both groups (Figure 2F). In the OFT, CORTI-Veh mice spent less time in the center than Sham-Veh mice, indicating increased anxiety. This anxiogenic effect of CORTI was rescued by SG2A administration (Figure 2G). Total activities were not significantly different among the experimental groups (Supplementary Figure 1C).

In the TST, the immobility of CORTI-Veh mice was significantly higher than that of Sham-Veh mice, indicating an increased depression-like behavior. SG2A reduced the immobility of both groups (Figure 2H).

The DG of the hippocampus is particularly sensitive to highly sustained corticosterone, showing altered neural death and adult neurogenesis. Furthermore, neurogenesis in the DG is highly associated with depression, as antidepressant drugs increase neurogenesis (Schmidt and Duman, 2007). Thus, proliferating neural progenitors in the DG were examined by counting BrdU^+^DCX^+^ cells. The number of double-positive cells was significantly decreased in CORTI-Veh mice compared to that in Sham-Veh mice. SG2A treatment significantly augmented the number of BrdU^+^DCX^+^ cells in both groups (Figure 2I).

Depression-like behavior reflects an underactivity of monoaminergic transmission. We examined protein levels of TPH, the rate-limiting enzyme for serotonin (5-HT) synthesis, as our data, presented below, suggested that SG2A activates 5-HT neurons in the dorsal raphe nucleus (DRN) but not dopaminergic neurons in substantia nigra (SN) and the ventral tegmental area (VTA). CORTI markedly lowered TPH levels compared to those of Sham-Veh mice. This decrease in TPH levels was rescued by SG2A (Figure 2J).

### Effects of SG2A on Fear Memory Consolidation and Extinction

**Figure 3:**
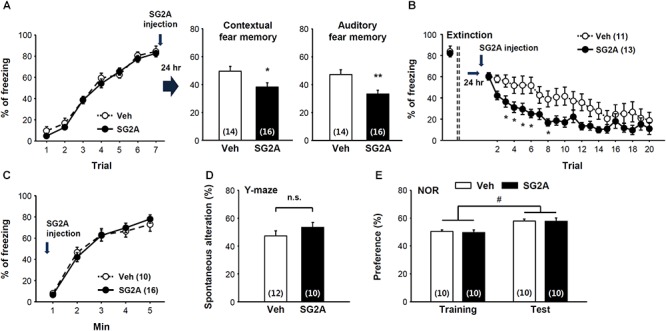
Effect of SG2A on fear memory consolidation and extinction. **(A)** Decreased fear memory consolidation by SG2A. Fear memory acquisition curves during seven repeated tones and foot shock pairing (left). SG2A was administered immediately after acquisition, and contextual fear memory (middle) and auditory fear memory (right) were determined 24 h later. **(B)** Facilitated extinction of consolidated fear memory by SG2A. 24 h after memory acquisition, freezing behaviors were scored during 20 trials of a 30-s tone without foot shock in the distinct context in the presence or absence of SG2A. **(C)** Innate fear response is unaltered by SG2A. Freezing behaviors of mice exposed to TMT were scored. **(D)** Recognition memory by SG2A in the Y maze. No significant difference (n.s.) was found between Veh- and SG2A-treated groups. **(E)** Novel object recognition test. Vehicle- and SG2A-treated mice similarly showed a preference for the novel object. Data are presented as means ± SEMs; ^∗^*P* < 0.05 and ^∗∗^*P* < 0.01 vs. Veh, #*P* < 0.05 between training and test group. Numbers in parentheses indicated the numbers of animals used for each group.

As SG2A exerted anxiolytic effects, we further determined the effects of SG2A on fear memory consolidation and extinction using the contextual and auditory-cued Pavlovian fear conditioning model. Seven trials of auditory cue with foot shock resulted in fear memory acquisition. Immediately after the last trial, SG2A or vehicle was administered into the LV of the mice (Figure 3A, left). The fear memory consolidation was determined 24 h later by placing the mice in the same chamber without auditory cues (context fear memory test) or in a different chamber with the auditory cues (auditory fear memory test). SG2A-administered mice spent significantly less time freezing than vehicle-treated mice in response to the context (Figure 3A, middle). Likewise, SG2A reduced the freezing rate responding to the auditory cue (Figure 3A, right). Thus, SG2A administration just after fear memory acquisition reduced both contextual and auditory fear memory consolidation after 24 h. Fear memory extinction was also examined at this time by determining the freezing rate in response to repeated auditory cues without foot-shock. SG2A or vehicle was administered 2–3 h before the first auditory cue. Vehicle-treated mice showed a gradual decrease in freezing rate as the number of trials increased, and SG2A accelerated this decrease beginning at the second trial [treatment effect: *F*(1,22) = 5.192, *P* < 0.05; interaction: *F*(1,22) = 1.773, *P* < 0.05] (Figure 3B). These results suggest that SG2A facilitates the extinction of fear memories. Whether these effects were due to a decreased response to fear-causing environments (unconditioned fear response) or to impaired recognition memory was not clear. However, SG2A did not alter unconditioned fear responses, as freezing behaviors of mice exposed to TMT, a synthetic fox (predator) feces odor, did not differ (Figure 3C). The results from the Y-maze test suggest the SG2A does not alter spatial recognition memory, as there was no difference between vehicle- and SG2A-injected mice (Figure 3D). Furthermore, SG2A did not influence responding in the NOR test (Figure 3E). Thus the effects of SG2A on fear memory were not attributable to a decreased response to a fear environment or an impairment of recognition memory.

### Neuronal Activation by SG2A

**Figure 4:**
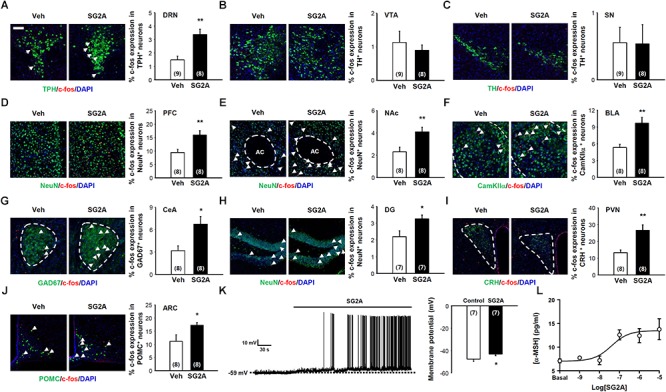
Neurons responding to SG2A. Immunohistochemistry for c-fos^+^ (red) neurons in mice administered SG2A or vehicle (Veh) for 1–2 h. The Percentage of cells double immunopositive for c-fos and TPH (serotonergic neurons) in the DRN **(A)**, TH (dopaminergic neurons) in the VTA **(B)** and SN **(C)**, NeuN in PFC **(D)** and NAc **(E)**, CamKIIα in BLA **(F)**, GAD67 in CeA **(G)**, NeuN in the DG of the hippocampus **(H)**, CRH in PVN **(I)**, and POMC in ARC **(J)** were counted (3–4 slices were counted per mouse). **(K)** Depolarization of POMC neuron membrane potential in response to applications of SG2A (left); summary of acute effects of SG2A on the membrane potentials of responsive POMC neurons (right). **(L)** α-MSH secretion in cultured POMC neurons in response to SG2A. Data are presented as means ± SEMs; ^∗^*P* < 0.05 and ^∗∗^*P* < 0.01 vs. Veh. Numbers in parentheses indicate the numbers of animals used for each group. Scale bar, 50 μm.

To determine the possible neural circuits for SG2A action, mRNA expression of SPX/GAL and GAL receptors were determined in the various brain regions using *in situ* hybridization assay and quantitative RT-PCR. SPX mRNA were strongly detected in the medial habenula (mHb) and suprachiasmatic nucleus (SCN) of the hypothalmus. Interestingly, GAL_2_ receptor mRNA levels were high in the hypothalamus, Hb, ventral midbrain (VMB), and DRN which are potential target regions of SPX action. In contrast, mRNA expressions of GAL, GAL_1_ receptor, and GAL_3_ receptor were mainly restricted in the hypothalamus (Supplementary Figure 2). To identify the neural circuits influenced by GAL_2_ receptor activation, the numbers of c-fos^+^ neurons in various brain regions were determined at 1–2 h after a single i.c.v. administration of SG2A, which is sufficient to induce anxiolytic-, antidepressant-, and anorexic-like effects in normal and ob/ob mice (Supplementary Figure 3). We found that SG2A significantly increased the number of c-fos^+^TPH^+^ 5-HT neurons in the DRN (Figure 4A) but not the number of c-fos and TH double-positive dopaminergic neurons in the VTA (Figure 4B) and SN (Figure 4C). SG2A administration also induced c-fos in the prefrontal cortex (PFC) (Figure 4D) and the nucleus accumbens (NAc) (Figure 4E), regions implicated in the pathophysiology of depression and innervated by 5-HT neurons from the DRN (Challis and Berton, 2015). c-fos expression was also increased in the basolateral (BLA) and central amygdala (CeA). In particular, the number of neurons immunoreactive for both c-fos and CamKIIα (Figure 4F) or GAD67 (Figure 4G) was higher in SG2A-injected mice than in vehicle-treated mice. In the hippocampus, SG2A increased the number of c-fos^+^ neurons in DG (Figure 4H) but not in CA1, CA2, or CA3 (Supplementary Figure 4). The activation of neurons in the amygdala and DG is implicated in regulating anxiety and fear memory (Sierra-Mercado et al., 2011). Increased c-fos immunoreactivity was also observed in CRH neurons in the paraventricular nucleus (PVN) (Figure 4I) and POMC neurons in the arcuate nucleus (ARC) (Figure 4J). However, other brain regions, such as Hb, lateral hypothalamus, and SCN, did not show c-fos induction after SG2A administration (Supplementary Figure 4).

As POMC neurons in the ARC are particularly important for regulating body weight via appetite and energy expenditure (Schwartz et al., 2000), we further examined whether SG2A can directly activate these neurons *ex vivo* and *in vitro*. POMC neurons in hypothalamic slices obtained from POMC-hrGFP transgenic mice can be identified by their green fluorescence (Sohn et al., 2011). As shown in Figure 4K, bath application of SG2A depolarized the membrane potentials of 7/18 (38.9 %) POMC neurons by 4.6 ± 0.6 mV (from -47.7 ± 2.1 to -43.1 ± 1.5 mV, *P* < 0.05). The other 11 cells remained unresponsive to SG2A (from -46.8 ± 2.2 to -46.6 ± 2.2 mV, *P* > 0.05). These data demonstrate that GAL_2_ receptor stimulation excites POMC neurons. *In vitro*, cultured POMC neurons exhibited dose-dependent SG2A-induced secretion of α-MSH, a major anorexic neuropeptide (Figure 4L; Yang and Tao, 2017).

### Nasal Application of SG2A

**Figure 5:**
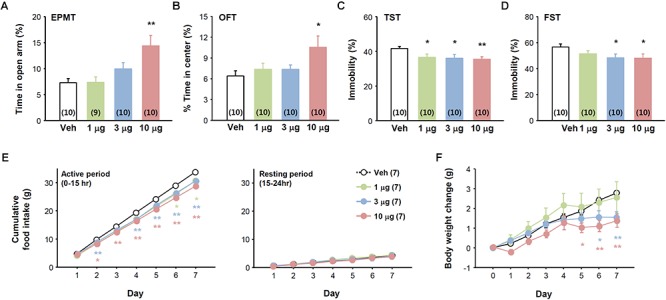
Effects of intranasally administered SG2A on anxiety, depression-like, and feeding behaviors. Normal mice received various concentrations of SG2A (1, 3, and 10 μg/mouse). Anxiolytic effect of SG2A in the EPMT **(A)** and OFT **(B)**. Antidepressant-like effect of SG2A in the TST **(C)** and FST **(D)**. Cumulative changes in food intake **(E)** and body weight **(F)** during 7 consecutive administrations of SG2A. Data are presented as means ± SEMs; ^∗^*P* < 0.05 and ^∗∗^*P* < 0.01 vs. Veh. Numbers in parentheses indicate the numbers of animals used for each group.

The effects of SG2A in these animal models suggest it has therapeutic potential. Thus, we examined whether SG2A can be delivered to the CNS by i.n. administration, a non-invasive route for enabling efficient crossing of the blood-brain barrier (Kageyama et al., 2016). Behavioral tests were performed 2–3 h after SG2A i.n. administration. Similar to that observed with i.c.v. administration, mice receiving i.n. SG2A spent more time in the open-arms in the EPMT (Figure 5A) and open field in OFT (Figure 5B) without significant changes in total movement (Supplementary Figure 5A) and significantly less immobility in the TST (Figure 5C) and FST (Figure 5D), suggesting that i.n. SG2A reduced anxiety- and depression-like behaviors. When administered for 7 days, i.n. SG2A significantly decreased cumulative food intake and body weight in a dose-dependent manner. SG2A decreased food intake during the active period when the mice mainly consume the chow, but not during the resting period (Figure 5E). These decreases corresponded to continual decreases in body weight, with a significant difference versus the vehicle-treated group observed beginning day 5 after SG2A administration for the 10 μg-treated group and day 6 for the 3 μg-treated group (Figure 5F).

Successful delivery of SG2A to the brain via the i.n. route was confirmed by c-fos induction in the brain regions that responded to i.c.v. administration. Specifically, i.n. administration of SG2A increased the numbers of c-fos^+^TPH^+^ 5-HT neurons in the DRN, c-fos^+^NeuN^+^ neurons in the PFC, NAc, and DG, c-fos^+^CamKIIα^+^ cells in the BLA, and c-fos^+^GAD67^+^ cells in the CeA (Supplementary Figure 5). Antidepressive and anxiolytic effects of SG2A i.n. administration were also examined in CORTI mice. Nasal delivery of SG2A increased the time in the open-arm in the EPMT, increased time in the center in OFT, and decreased the time of immobility in the TST without any differences in total activities (Supplementary Figure 6). Altogether, these results demonstrate that SG2A can be delivered to the CNS via an i.n. route to sufficiently evoke neurochemical and behavioral effects similar to those observed after i.c.v. administration.

## Discussion

Considering the cross-reactivity of SPX and GAL toward GAL receptors and the overall complexity of the GAL receptor subtype-specific signaling pathways (Kim et al., 2014; Yun et al., 2014), the development of subtype-selective GAL receptor agonists may help to elucidate GAL receptor subtype-specific physiological functions (Reyes-Alcaraz et al., 2016). Many different types of GAL receptor-targeting agonists/antagonists have been generated by using a variety of fragments from the GAL peptide. However, these agents maintained their substantial affinity toward other GAL receptor subtypes at high concentrations (Webling et al., 2012). Recently, we developed SG2A, an SPX-based GAL_2_ receptor agonist that lacks activity toward GAL_1_ receptor and GAL_3_ receptor (Reyes-Alcaraz et al., 2016). The pharmacological relevance of SG2A is underscored by its biased agonism toward G-protein-dependent signaling over β-arrestin-mediated signaling, which is similar to SPX but different from GAL with unbiased activation (Reyes-Alcaraz et al., 2018). Probably due to β-arrestin-mediated signaling, classical agonists often result in contradictory results, such as with GAL for depression-like behaviors (Lu et al., 2005; Kuteeva et al., 2008) and the dichotomous action/dose-dependent inaction of galanin-like peptide in regulating food intake (Lawrence et al., 2002; Kageyama et al., 2016). By contrast, SG2A shows dose-dependent and consistent effects on mood and appetite behaviors during repetitive administrations. Thus, compared to the GAL-based agonist, the SPX-based agonist may have multiple pharmacological benefits, exerting its action on both mood and appetite/body weight.

SG2A administration (i.c.v./i.n.) produced rapid anxiolytic- and antidepressant-like behavioral effects. Thus, time required for onset of effects is different from selective serotonin reuptake inhibitors (SSRIs) that show a late onset of antidepressive effects. Nevertheless, the effect of SG2A is likely mediated by activation of 5-HT neurons in the DRN as shown in Figure 4A. It is of interesting to note that *SPX* mRNA is highly expressed in the mHb. Projection from mHb to interpeduncular nucleus (IPN) is connected to the serotonergic raphe nucleus which is implicated in the regulation of mood such as depression and anxiety (Yamaguchi et al., 2013). In addition, the DRN is known to express GAL_2_ receptor which then promote TPH transcription (Xu et al., 1998; Lu et al., 2005; Mazarati et al., 2005). Indeed, our study showed that repetitive SG2A administration significantly increased TPH protein levels in the DRN of CORTI mice. Interestingly, chronic treatment of fluoxetine, an SSRI, increases GAL_2_ receptor binding sites on the DRN (Lu et al., 2005). Thus, both SG2A and SSRI are involved in direct or indirect activation of GAL_2_ receptor for increased activities of 5-HT neurons in the DRN. In addition, SG2A, similar to SSRIs, increases neurogenesis in the DG of the hippocampus. The hippocampal neurogenesis is decreased by inescapable stress events resulting in a state of behavioral despair and this effect can be reversed by fluoxetine (Malberg and Duman, 2003). Disruption of fluoxetine-induced neurogenesis suppresses behavioral responses to antidepressants, therefore the increased neurogenesis is likely an essential process for antidepressive behavioral effects (Santarelli et al., 2003). Thus, increase in c-fos^+^TPH^+^ 5-HT neurons by SG2A and similarity of histochemical changes in the brain between SSRI and SG2A may suggest that antidepressive and anxiolytic effects of SG2A in CORTI mice are mainly mediated by activation of 5-HT neurons in the DRN.

One of the unexpected side effects of SSRIs is body weight changes (Levine et al., 1987; Uguz et al., 2015). A recent study showed that long-term treatment of escitalopram, an effective SSRI antidepressant that is associated with significant weight gain (Uguz et al., 2015), led to the downregulation of SPX expression in the rat hypothalamus. This suggests that the increased body mass by SSRI may be, at least in part, due to a decreased anorexic action of SPX in the hypothalamus (Palasz et al., 2016). Both POMC and CRH neurons in the hypothalamus are pivotal neuronal populations involved in appetite/body weight regulation (Schwartz et al., 2000). In goldfish, i.c.v. administration of SPX inhibited food intake accompanying increased mRNA levels of POMC and CRH (Wong et al., 2013). Our results also showed that SG2A reduced food consumption with induced c-fos expression in POMC- and CRH-positive neurons. As SG2A reduced body weight in normal and obese mice, SG2A treatment can circumvent SSRI-induced increase in body weight by direct activation of anorexic neuronal population in the hypothalamus. POMC and CRH neurons in the hypothalamus sense and integrate inputs not only from peripheral tissues (Schwartz et al., 2000) but also central inputs from brain areas involved in stress/mood/hedonic/reward control (Adam and Epel, 2007; Carroll et al., 2012). Moreover, hedonic or reward-based control can override homeostatic pathway for eating behaviors and body weight controls. In CORTI mice that already lost body weight, SG2A increased their body weight, likely via relieving anhedonia, anxiety, and depression. Thus, it seems likely that the antidepressive effects of SG2A overwhelms the anorexic effects of SG2A in CORTI mice. Together, SPX and GAL_2_ receptor are likely connected to the key regulatory system controlling or linking appetite and mood behaviors, allowing optimal treatment for comorbid mood disorders and abnormal body weight.

Post-traumatic stress disorder (PTSD) is a severe anxiety disorder that involves an explicit conditioning episode (Dohrenwend et al., 2006; de Vries and Olff, 2009). Furthermore, PTSD and depression are commonly co-occurring mental disorders which reinforces each other. PTSD is frequently conceptualized as a memory disorder within a Pavlovian fear conditioning and extinction framework (Rubin et al., 2008; Shin and Handwerger, 2009). This study also showed that SG2A contributes to fear memory consolidation and extinction in a PTSD animal model. Specifically, SG2A decreased both hippocampus-dependent contextual and amygdala-dependent auditory fear responses and facilitated fear extinction after fear memory acquisition. Accordingly, SG2A administration activated neurons in the amygdala, PFC, and DG which are major components of the neural circuits that regulate anxiety and fear memory (Sierra-Mercado et al., 2011). Although there are reports of impaired cognition induced by GAL (Rustay et al., 2005), SG2A did not induce memory deficits in the Y-maze and NOR tests in this study.

A major challenge for treatments involving peptide drugs is bypassing the blood-brain barrier. Over the last several decades, diverse formulations and devices have been developed to transport the drugs from the nose directly to the brain, showing promise for therapeutic efficacy based on animal models and clinical trials in humans (Craft et al., 2012; Striepens et al., 2013; Kageyama et al., 2016). Notably, i.n. administrations of GLP2, oxytocin, or other peptides produce antidepressive effects or improve social behavior, thereby attracting attention to the development of peptide drugs for the treatment of neuropsychiatric diseases (Sasaki-Hamada et al., 2017). The results of this study showed that SG2A can be successfully delivered to the CNS via the i.n. route, producing results comparable to those obtained with i.c.v. delivery.

## Conclusion

In conclusion, SG2A exerts a rapid onset of effects toward relieving anxiety-, depression-like, and feeding behaviors and suppressing fear memory. These effects were maintained during repetitive SG2A administrations delivered either i.c.v./i.n. SG2A has potential for the clinical application to treat mood disorders and/or abnormal appetite/body weight.

## Data Availability

All datasets generated for this study are included in the manuscript and/or the Supplementary Files.

## Ethics Statement

All animal procedures were approved by the Institutional Animal Care and Use Committee of Korea University (KOREA-2016-0050-C2) and Korea Advanced Institute of Science and Technology (KA2014-02).

## Author Contributions

SY, AR-A, B-JH, J-WS, D-HK, GS, HK, S-GK, DK, and BK designed the research. SY, AR-A, Y-NL, HY, and JC performed the research. SY and AR-A analyzed the data. J-IH and JS wrote the manuscript. All authors read and approved the final manuscript.

## Conflict of Interest Statement

SY, AR-A, Y-NL, HY, GS, J-IH, and JS are shareholders. S-GK and DK are employees and shareholders. BK is a shareholder and the CEO of Neuracle Science Co., Ltd. The remaining authors declare that the research was conducted in the absence of any commercial or financial relationships that could be construed as a potential conflict of interest.
