# Supplementary material for: Spexin-Based Galanin Receptor Type 2 Agonist for Comorbid Mood Disorders and Abnormal Body Weight
Source: Front Neurosci. 2019 Apr 18;13:391. doi: 10.3389/fnins.2019.00391 (PMC6482256; doi:10.3389/fnins.2019.00391)
Supplement: Supplementary file 1 [file Data_Sheet_1.PDF]

# Supplementary Information

## Supplementary Materials and Methods

### Behavioral studies

*Body weight and food intake measurements.* Body weight and food intake were monitored daily throughout the experiment. Body weights were measured in the morning and food intake was measured twice a day (15 and 24 h after SG2A administration).

*Elevated plus maze test (EPMT).* The maze consisted of two open and two closed arms (5 × 30 cm each) with 20-cm-high walls. The apparatus was elevated 50 cm above the ground. Mice were individually placed in the center of the apparatus facing an open arm and allowed to explore for 10 min. The frequency and duration of arm entries were recorded. An entry was defined as the movement of all four paws into an arm. The percentage of time spent in open arms was scored (Chung et al., 2014).

*Open filed test (OFT).* The OFT apparatus consisted of a box (40 × 40 × 40 cm) of opaque gray plastic. Mice were placed in the center of the box, and their behavior during a 5-min test period was tracked by a video camera positioned above the center of the apparatus. The frequency of entering and percentage of time spent in the central area (central 25% of the floor) and the total distance traveled were scored (Chung et al., 2014).

*Depression-like behaviors.* The despair-based tail suspension test (TST) and the FST were performed as previously described (Chung et al., 2014). For the TST, the mice were individually suspended by their tails in a white box (36.5 × 30.5 × 30.5 cm) for 6 min. They

were considered immobile when agitation and escape attempts ceased. For the FST, the mice were placed in a transparent beaker (10-cm diameter) containing water (21¯25°C) at a depth of 15 cm for 6 min. Immobility, defined as the absence of volitional body or limb movement, was scored as the percentage of time spent immobile during the last 4 min of the test.

*Sucrose preference test (SPT).* The SPT was conducted according to previously described procedures with a minor modification (Shin et al., 2015). The mice were singly housed and given access to two bottles (containing water or 2% sucrose in water) for 2 days. The positions of the water and sucrose bottles were switched daily to ensure that the mice did not develop a side preference. The SPT procedure started before lights off and finished after lights on. Sucrose preference was calculated as a percentage of the amount of sucrose consumption (100 × volume from sucrose-containing bottle/total volume consumption from both bottles).

*Pavlovian fear conditioning*. Mice were first habituated for 10 min in the conditioning chamber (18 × 18 × 30 cm) without disturbance. The next day, the mice were placed in the conditioning chamber, and seven conditioning trial repetitions, each consisting of a tone (30 s, 5 kHz, 75 dB) that terminated with foot shocks (0.7 mA, 2 s) with a 60-s intertrial interval, were administered. Conditioned fear responses were tested the following day by placing the mice in the same chamber (without foot shock) and measuring the freezing time over a period of 5 min. For the auditory test, mice were placed in a distinct context and re-exposed to three tones without foot shock at 90-s intervals after a 5-min period of exploration. Freezing behaviors were scored during the tone presentation. The total freezing time in the test period was represented as a percentage of the average duration to each tone presentation. Conditioned freezing was defined as immobility except for respiratory movements (Lee et al.,

2011). During extinction training, the mice were exposed to 20 trials of a 30-s tone without any foot shock in the distinct context.

*Unconditioned innate fear response.* Mice were placed in a chamber containing 30 µl of synthetic predator fox feces odor ([TMT] 2,5-dihydro-2,4,5-trimethylthiazoline, $C_6H_{11}NS$; SRQ Bio, Sarasota, FL). TMT-evoked freezing behavior was recorded for 15 min and scored as the average percentage of freezing time from 3-min intervals (Lee et al., 2011).

*Y maze test.* The Y maze test was conducted according to previously described procedures with a minor modification (Dellu et al., 1992). The Y maze had three identical arms (30 × 5 cm) with 20-cm-high walls. The mice were placed in the center of the maze and the sequence of arm entries over 5 min was recorded. The percentage alteration was calculated as the number of triads containing entries into all three arms (ABC, ACB, BAC, BCA, CAB, CBA) divided by the maximum possible alterations (equivalent to the total number of arms entered - 2) × 100.

*Novel object recognition (NOR) test.* Mice were first allowed to freely explore the open field (40 × 40 × 40 cm) for 10 min for habituation. The next day, the mice were returned to the open field that had two identical objects placed 5 cm apart from the wall of open field, and video was recorded for 10 min. Twenty-four hours later, the mice were returned to the box with one of the familiar objects replaced by a novel object. The open field and the objects were cleaned with 70% ethanol and dried before each use to minimize error due to olfactory cues. The time spent toward each object was recorded (Leger et al., 2013).

**Western blotting**

Brain tissues were lysed with a buffer containing 50 mM Tris-HCL (pH 7.5), 0.1% sodium dodecyl sulfate, and protein inhibitor cocktails (Roche Applied Science, Penzberg, Germany). Protein contents in the lysates were quantified with Bradford protein assay reagent (Bio-Rad, Hercules, CA). Proteins (25–30 µg) were resolved on sodium dodecyl sulfate-polyacrylamide gels and transferred to nitrocellulose blotting membranes in a Trans-Blot electrophoresis apparatus (Bio-Rad). The blots were blocked in Tris-buffered saline containing 0.3% Tween 20 and 5% skim milk and incubated with a primary antibody for TPH (Sigma-Aldrich) overnight at 4℃. They were then washed three times with Tris-buffered saline with 0.3% Tween 20. The blots were incubated with horseradish peroxidase-conjugated secondary antibodies (Jackson ImmunoResearch Laboratories, West Grove, PA). The blots were washed three times and immunoreactive bands were visualized by exposure to X-ray film for 0.5 or 10 min, after applying enhanced chemiluminescence reagents (GE Healthcare, Chicago, IL).

**Electrophysiology**

Whole-cell patch-clamp recordings from POMC neurons maintained in hypothalamic slice preparations and data analysis were performed as previously described. Briefly, 6- to 9-week-old POMC-hrGFP transgenic mice were anesthetized and transcardially perfused with a modified ice-cold artificial cerebrospinal fluid (ACSF), in which an equimolar amount of sucrose was substituted for NaCl. The mice were then decapitated, and the entire brain was removed and immediately submerged in ice-cold carbogen-saturated (95% $O_2$ and 5% $CO_2$) ACSF (126 mM NaCl, 2.8 mM KCl, 1.2 mM $MgCl_2$, 2.5 mM $CaCl_2$, 1.25 mM $NaH_2PO_4$, 26 mM $NaHCO_3$, and 5 mM glucose). Coronal sections (250 µm) were cut from a brain block containing the hypothalamus with a Leica VT1200S Vibratome and then incubated in oxygenated ACSF at 34°C for at least 1 h. The slices were then transferred to the recording

chamber and allowed to equilibrate for 10–20 min in oxygenated ACSF (32–34°C) at a flow rate of ~3 ml/min. The pipette solution for whole-cell recording was modified to include an intracellular dye: 120 mM K-gluconate, 10 mM KCl, 10 mM HEPES, 5 mM EGTA, 1 mM $CaCl_2$, 1 mM $MgCl_2$, 2 mM MgATP, and 0.3 mM NaGTP, 0.03 mM Alexa Fluor 594 hydrazide dye (pH 7.3). Epifluorescence was briefly used to target fluorescent cells, at which time the light source was switched to infrared differential interference contrast imaging to obtain the whole-cell recording (optiMOS; QImaging, Surrey, Canada). Electrophysiological signals were recorded using an Axopatch 700B amplifier (Molecular Devices, San Jose, CA), low-pass filtered at 1 kHz, and analyzed with pCLAMP programs (Molecular Devices). Recording electrodes had resistances of 2.5–5 MΩ when filled with the K-gluconate internal solutions. Membrane potential values were not compensated to account for junction potential (-8 mV). For some experiments SG2A (1 μM) in ACSF was perfused for 4–6 min. A drug effect was required to be associated temporally with peptide application, and the response had to be stable within a few minutes. A neuron was considered depolarized or hyperpolarized if the membrane potential changed at least 2 mV in amplitude.

**Determination of α-MSH secretion in POMC neurons**

α-MSH secretion was determined using an adult mouse POMC cell line (CLU-500; Cellutions Biosystems Inc., Toronto, Canada) and a radioimmunoassay kit (RK-043-01; Phoenix Pharmaceuticals Inc., Burlingame, CA) according to the manufacturer's instructions. Briefly, POMC neurons were seeded in 6-well plates at a density of $6 \times 10^5$ cells/well in low-glucose DMEM. After 18 h, the cells were treated for 40 min with 1 ml of a solution containing SG2A. One hundred microliters from each well was incubated with 100 μl of α-MSH antibody and 100 μl of $^{125}$I-peptide for 24 h at 4°C. Next, 100 μl each of goat-anti-

rabbit IgG and normal rabbit sera were added, gently vortexed, and incubated for 90 min at room temperature. All tubes were centrifuged, the supernatants were carefully aspirated, and the cpm values of the pellets were measured with a γ counter (Wizard 1470-020; Perkin Elmer Wallac, Waltham, MA). The results were calculated according to the manufacturer's instructions.

**In situ hybridization**

Adult mice and rats were sacrificed, and mouse brains were removed and quickly frozen in isopentane on dry ice. Tissue sections were cut to 20 μm thickness with a cryostat, thaw mounted on Superfrost Plus slides (Thermo Fisher Scientific, USA), and stored at −70°C until use. Sections were fixed in 4% paraformaldehyde (PFA), washed with phosphate-buffered saline (PBS), and acetylated with 0.25% acetic anhydride in 0.1 M triethanolamine/0.9% NaCl (pH 8.0). Samples were hybridized overnight with a radiolabeled probe (1.2 × 106 cpm) and washed four times with 2× standard sodium citrate (SSC). A template for the SPX probe was prepared by subcloning the RT-PCR products into a pGEM-T vector (Promega). Following primers were used for the preparation of radio-labeled mouse SPX cDNA probe, upper: gtgcaccgggaagggaatg, lower: gttagtctccttggtatagg (the product size, 453 bps). Sense and antisense riboprobes were prepared using an in vitro transcription system (Promega) in the presence of [α-35S] UTP (Amersham Pharmacia Biotech, USA). After RNase A treatment, slides were rinsed with 2×, 1×, 0.5×, and 0.1× SSC containing 1 mM dithiothreitol for 10 min each at room temperature, then washed with 0.1× SSC at 60°C. The samples were dehydrated in ethanol and exposed to X-ray film (Biomax MR, Kodak, USA).

**RNA isolation and quantitative RT-PCR**

Total RNA from tissues was isolated by single-step acid guanidinium thiocyanate-phenol-chloroform method. For RT-PCR 500 ng of each RNA sample was reverse-transcribed with MMLV reverse transcriptase (Promega, Madison, WI). Then aliquots of the cDNA were subjected to quantitative real-time PCR in the presence of SYBR I (Sigma, St. Louis, MO). Gene expression levels were normalized with glyceraldehyde 3-phosphate dehydrogenase (GAPDH). Quantitative real-time PCR was carried out with the following primers: galanin, 5'-GCC CAC ATG CCA TTG ACA AC-3', 5'-GCG GAC AAT GTT GCT CTC AG-3'; SPX, 5'-CCC AGA TCT TGA ACT GCT GA-3', 5'-TTT CCA AGG AAG CCA GAA AC-3'; $GAL_1$ receptor, 5'-TGT CTG TGG ATC GCT ACG TG-3', 5'-ATG GAA AAG ACG CTG GTG GT-3'; $GAL_2$ receptor, 5'-CCC TCG ATC CAG CCT GTT AAA-3', 5'-CAT GAC CCC CAG GTA TCG TC-3'; $GAL_3$ receptor, 5'-TGC AAG ACG GTA CAT CTG CT-3', 5'-CGC CAG GTA CTA TCC ACT GA-3', GAPDH, 5'-CAT CCA CTG GTG CTG CCA AGG CTG T-3', 5'-ACA ACC TGG TCC TCA GTG TAG CCC A-3'.

## Supplementary Figures and Figure Legends

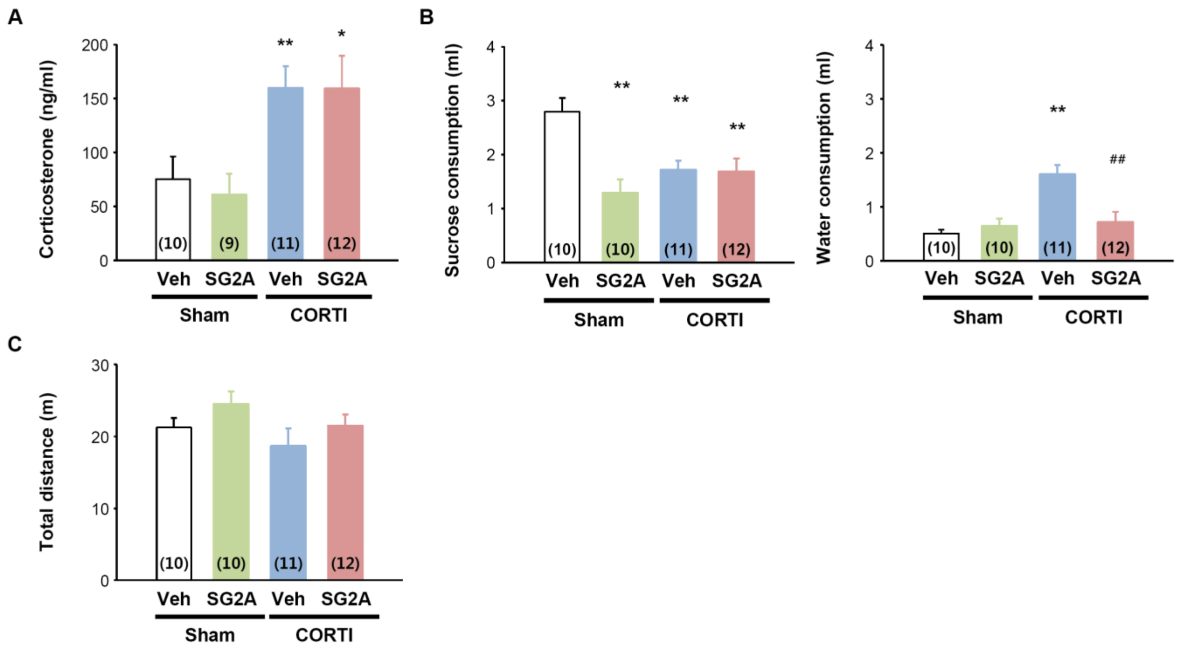


**Supplementary Figure 1.** Effects of SG2A in CORTI mice. **(A)** Sustained high level of corticosterone in mice with corticosterone pellet implantation (CORTI). Effects of SG2A on sucrose and water consumption in the sucrose preference test **(B)** and total movement in the open field test **(C)**. Data are presented as means ± SEMs; $^{**}P < 0.01$ vs. Sham-Veh, $^{\#\#}P < 0.01$ vs. CORTI-Veh. Numbers in parentheses indicate the numbers of animals used for each group.

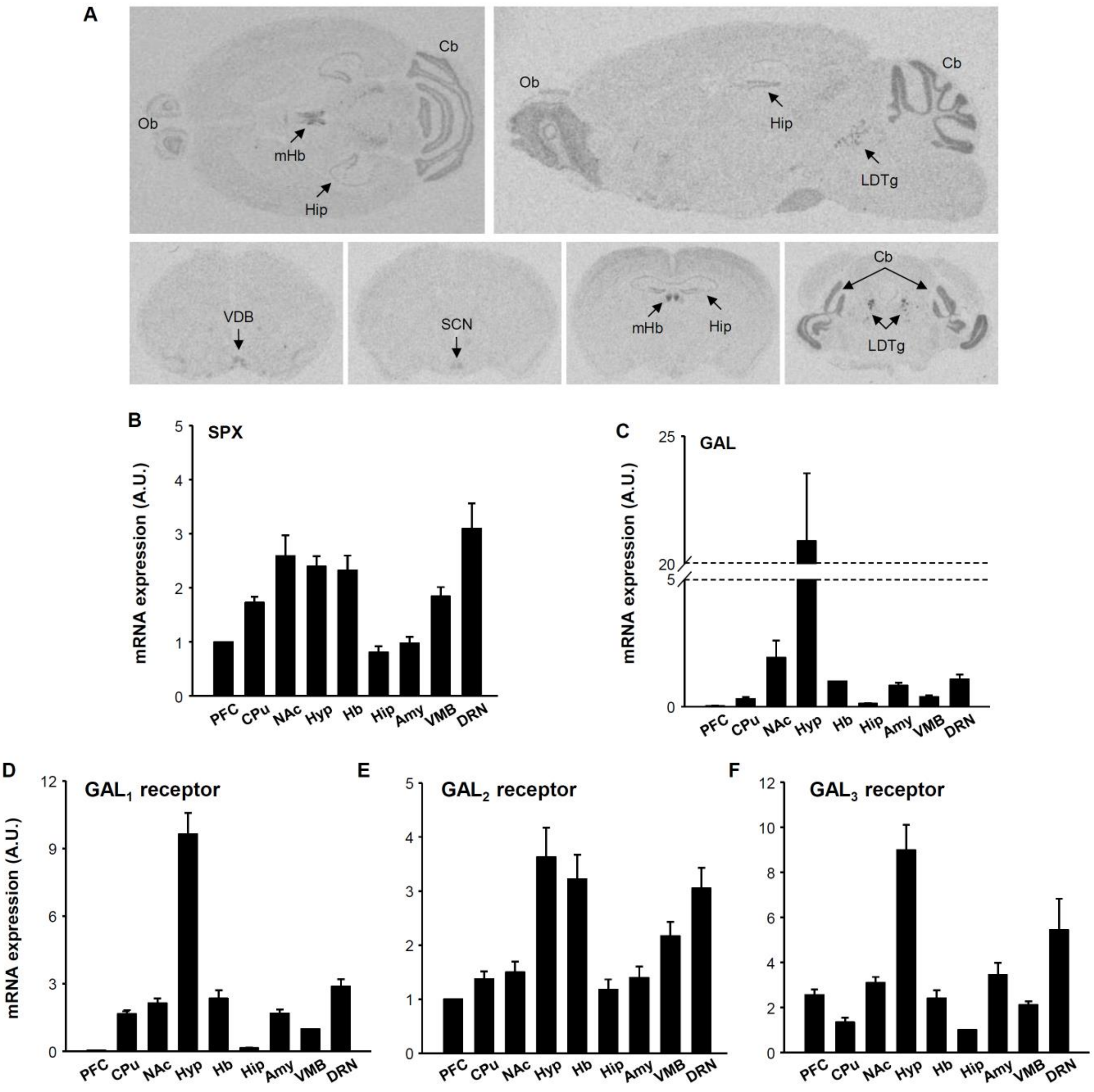


**Supplementary Figure 2.** Expression patterns of SPX/GAL peptides and $GAL_{1,2,3}$ receptor. **(A)** mRNA expression of SPX was determined by in situ hybridization. **(B-F)** mRNA levels of SPX **(B)**, GAL **(C)**, $GAL_1$ receptor **(D)**, $GAL_2$ receptor **(E)**, $GAL_3$ receptor **(F)** were determined by quantitative RT-PCR. The expression levels were normalized with GAPDH and expressed as mean ± SEMs. Expression value in PFC for SPX and $GAL_2$ receptor (B and E), Hb for GAL (C), VMB for $GAL_1$ receptor (D), and Hip for $GAL_3$ receptor (F) was set at 1. Abbreviations: Amy, amygdala; Cb, cerebellum; CPu: caudate putamen; DRN, dorsal raphe nucleus; Hb, habenula; Hip, hippocampus; Hyp, hypothalamus; LDTg, laterodorsal tegmental nucleus; mHb, medial habenula; NAc, nucleus accumbens; Ob, olfactory bulb; PFC: prefrontal cortex; SCN, suprachiasmatic nucleus; VDB, vertical diagonal band; VMB, ventral midbrain.

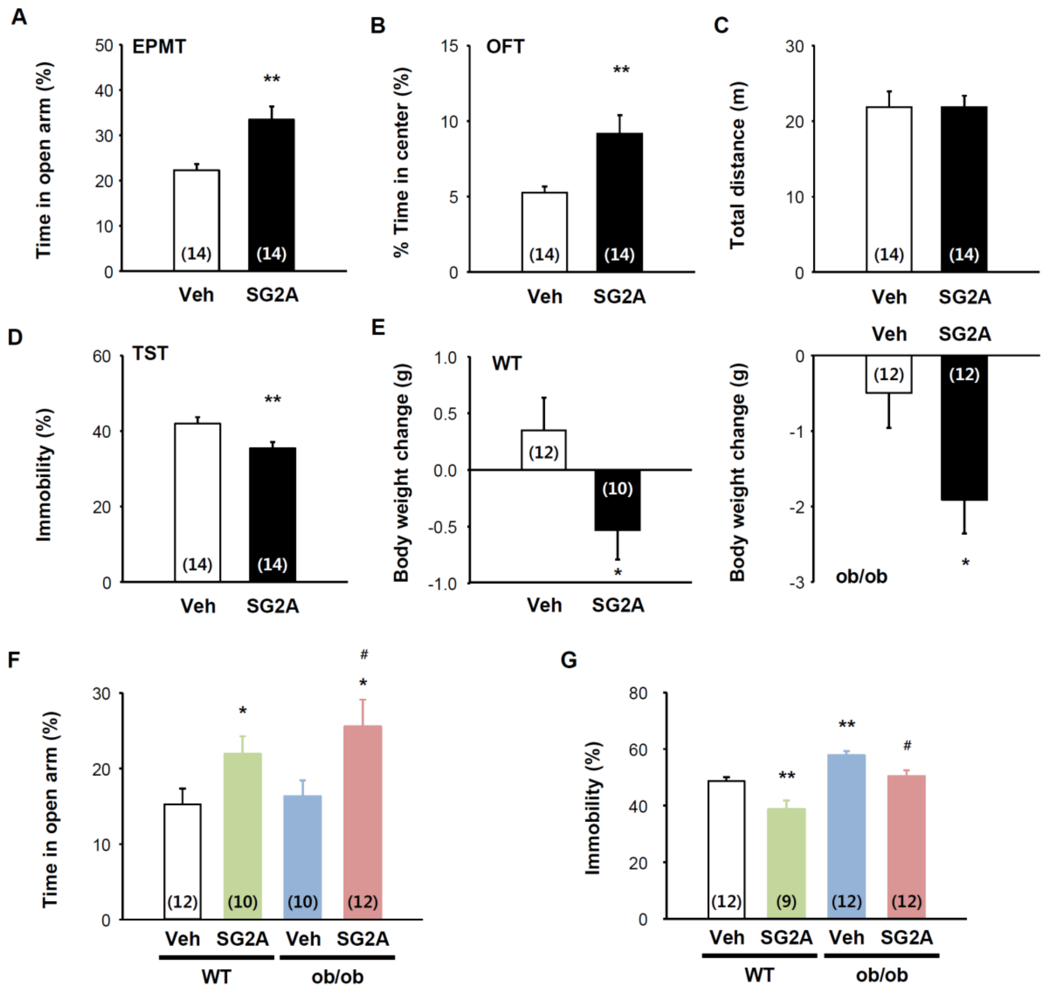


**Supplementary Figure 3.** Acute effects of SG2A administration and effect of SG2A in ob/ob mice. Effects of a single administration of SG2A on the time spent in the open arms in the elevated plus maze test **(A)** and the time spent in the center **(B)** and total movement **(C)** in the open field test. **(D)** Effect of SG2A on the immobility of mice in the tail suspension test. **(E)** Effect of SG2A in bodyweight changes in wild-type (WT) and leptin knockout (ob/ob) mice. Data are presented as means ± SEMs, *$P < 0.05$ and **$P < 0.01$ vs. Veh.

Anxiolytic and anti-depressive effects of SG2A in ob/ob mice on the time spent in the open arms in the elevated plus maze test **(F)** and the time spent in the center **(G)**. Data are presented as means ± SEMs, *$P < 0.05$ and **$P < 0.01$ vs. WT-Veh, $^{\#}P < 0.05$ vs. ob/ob-Veh. Numbers in parentheses indicate the numbers of animals used for each group.

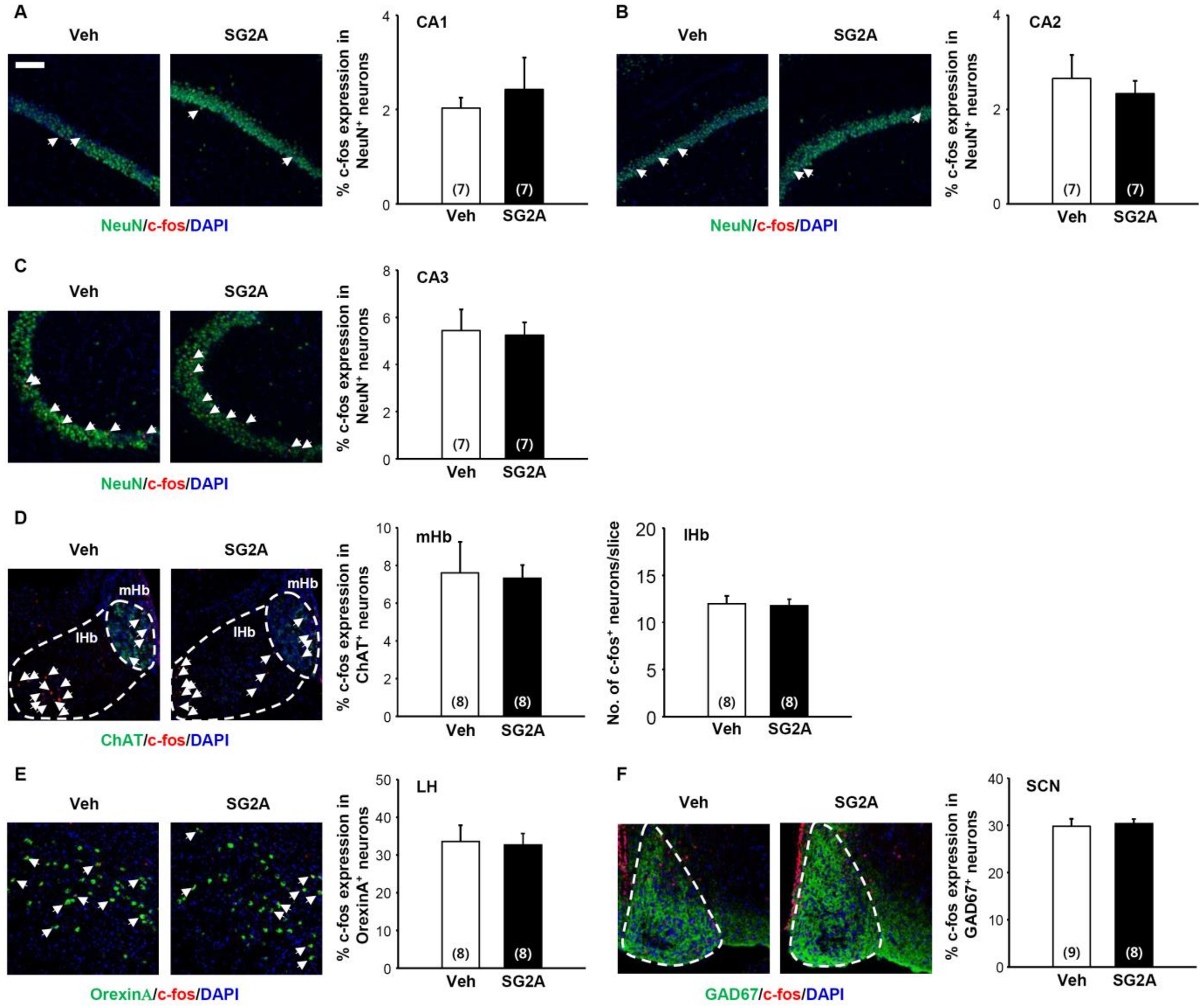


**Supplementary Figure 4.** Brain regions where SG2A did not induce c-fos expression. Immunohistochemistry for c-fos$^{+}$ (red) neurons in mice administered SG2A or vehicle (Veh) for 1–2 h. The numbers of cells double immunopositive for c-fos and NeuN in the CA1 **(A)**, CA2 **(B)**, and CA3 **(C)**, ChAT in medial habenula (mHb) and lateral habenula (lHb) **(D)**, orexinA in lateral hypothalamus (LH) **(E)**, and GAD67 in suprachiasmatic nucleus (SCN) **(F)** were counted (3–4 slices were counted per mouse). Data are presented as means ± SEMs. Numbers in parentheses indicate the numbers of animals used for each group. Scale bar, 50 µm.

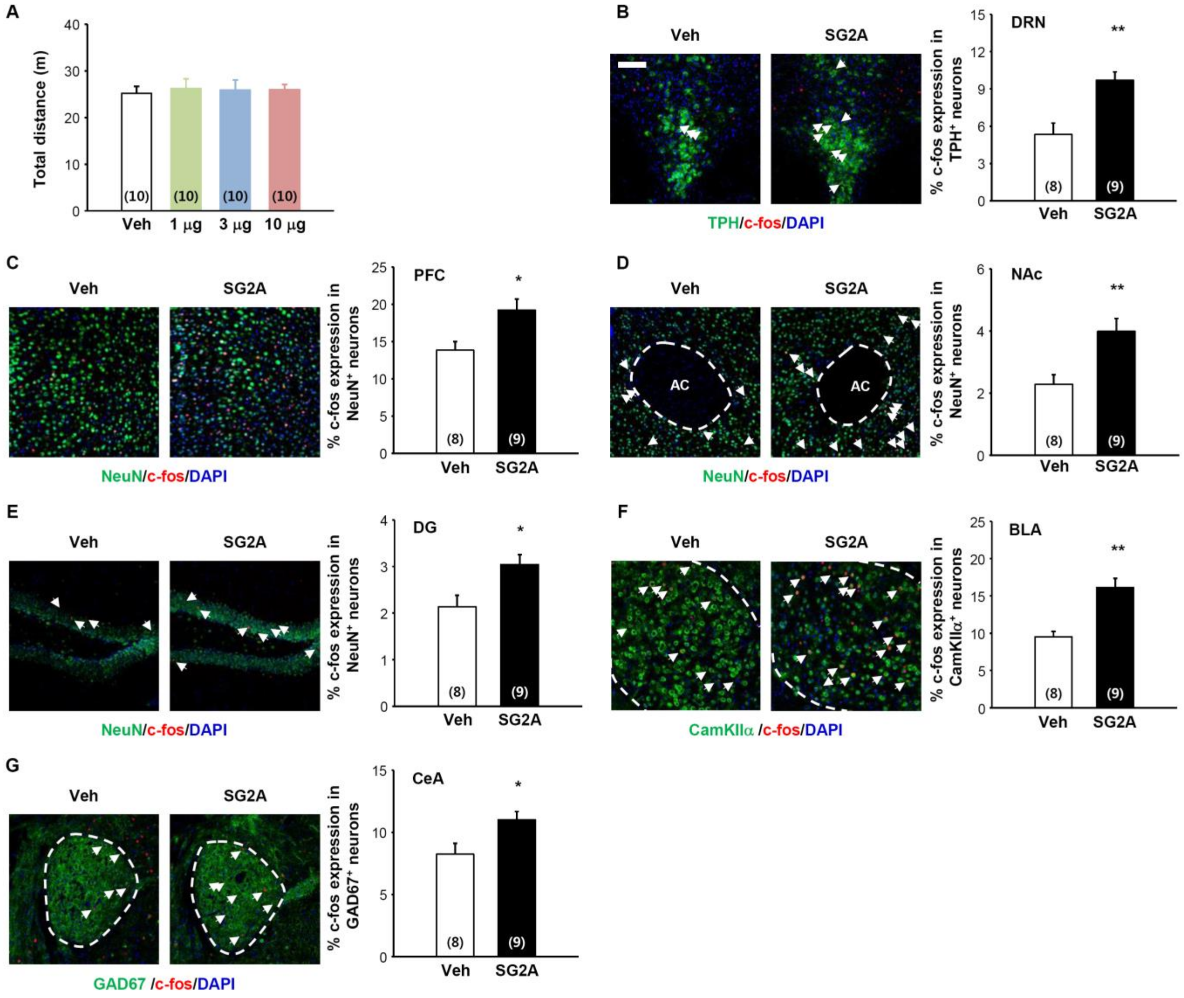


**Supplementary Figure 5.** Effects of SG2A intranasal (i.n.) administration. Effects of a i.n. administration of SG2A on total movement in the open field test **(A)**. Neurons responding to SG2A via intranasal administration **(B-G)**. Immunohistochemistry for c-fos$^+$ (red) neurons in mice administered SG2A or vehicle (Veh) for 2–3 h. The numbers of cells double immunopositive for c-fos and TPH in the DRN **(B)**, NeuN in PFC **(C)**, NAc **(D)**, and DG of hippocampus **(E)**, CamKIIα in BLA **(F)**, and GAD67 in CeA **(G)** were counted (3–4 slices were counted per mouse). Data are presented as means ± SEMs, $*P < 0.05$ and $**P < 0.01$ vs. Veh. Numbers in parentheses indicate the number of animals used for each group. Scale bar, 50 μm.

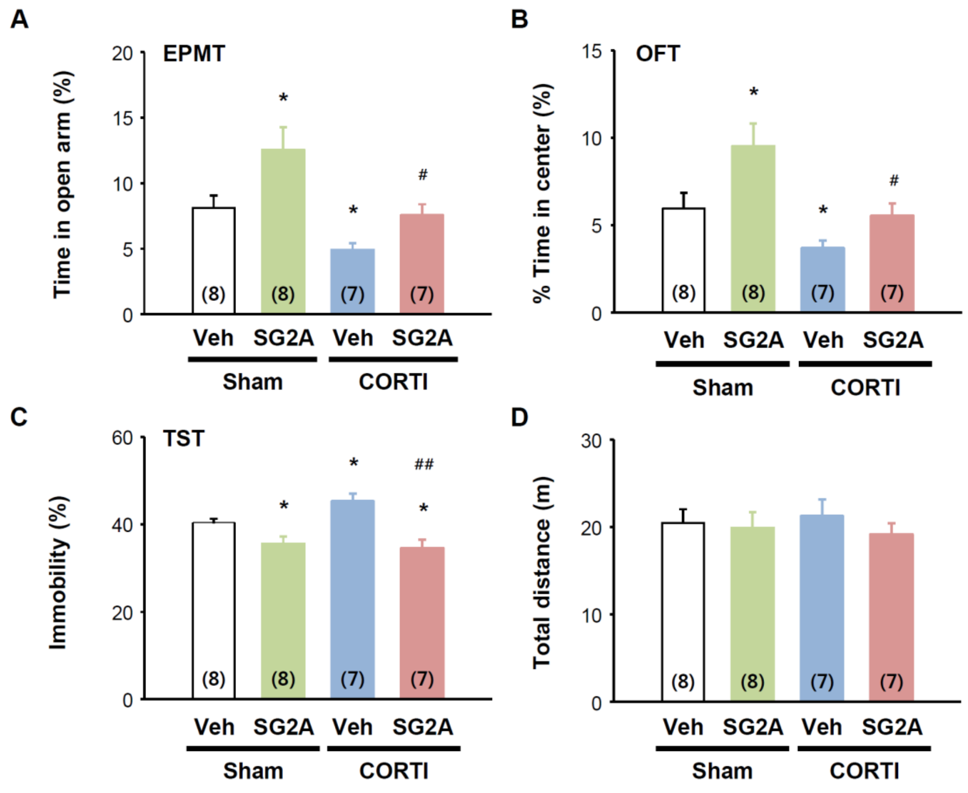


**Supplementary Figure 6.** Antidepressive and anxiolytic effects of SG2A i.n. administration in CORTI mice. Effects of SG2A i.n. administration in the times in the open-arm in the EPMT (A), and in the center in the OFT (B), and immobility of mice in the TST (C), and total movement (D). Data are presented as means ± SEMs; $*P < 0.05$ vs. Sham-Veh, $^{\#}P < 0.05$ and $^{\#\#}P < 0.01$ vs. CORTI-Veh. Numbers in parentheses indicate the numbers of animals used for each group.

## Supplementary References


Chung, S., Lee, E.J., Yun, S., Choe, H.K., Park, S.B., Son, H.J., et al. (2014). Impact of circadian nuclear receptor REV-ERBalpha on midbrain dopamine production and mood regulation. *Cell* 157, 858-868. doi: 10.1016/j.cell.2014.03.039.

Dellu, F., Mayo, W., Cherkaoui, J., Le Moal, M., and Simon, H. (1992). A two-trial memory task with automated recording: study in young and aged rats. *Brain Res.* 588, 132-139.

Lee, E.J., Son, G.H., Chung, S., Lee, S., Kim, J., Choi, S., and Kim, K. (2011). Impairment of fear memory consolidation in maternally stressed male mouse offspring: evidence for nongenomic glucocorticoid action on the amygdala. *J. Neurosci.* 31, 7131-7140. doi: 10.1523/JNEUROSCI.4692-10.2011.

Leger, M., Quiedeville, A., Bouet, V., Haelewyn, B., Boulouard, M., Schumann-Bard, P., and Freret, T. (2013). Object recognition test in mice. *Nat. Protoc.* 8, 2531-2537. doi: 10.1038/nprot.2013.155.

Shin, S., Kwon, O., Kang, J.I., Kwon, S., Oh, S., Choi, J., et al. (2015). mGluR5 in the nucleus accumbens is critical for promoting resilience to chronic stress. *Nat. Neurosci.* 18, 1017-1024. doi: 10.1038/nn.4028.
